# An *In Silico* Modeling Approach to Understanding the Dynamics of Sarcoidosis

**DOI:** 10.1371/journal.pone.0019544

**Published:** 2011-05-27

**Authors:** Baltazar D. Aguda, Clay B. Marsh, Michael Thacker, Elliott D. Crouser

**Affiliations:** 1 Neuro-Oncology Branch, Center for Cancer Research, National Cancer Institute, Bethesda, Maryland, United States of America; 2 Division of Pulmonary, Allergy, Critical Care, and Sleep Medicine, The Ohio State University Medical Center, Columbus, Ohio, United States of America; 3 Department of Electrical and Computer Engineering, The Ohio State University Medical Center, Columbus, Ohio, United States of America; University of Pittsburgh, United States of America

## Abstract

**Background:**

Sarcoidosis is a polygenic disease with diverse phenotypic presentations characterized by an abnormal antigen-mediated Th1 type immune response. At present, progress towards understanding sarcoidosis disease mechanisms and the development of novel treatments is limited by constraints attendant to conducting human research in a rare disease in the absence of relevant animal models. We sought to develop a computational model to enhance our understanding of the pathological mechanisms of and predict potential treatments of sarcoidosis.

**Methodology/Results:**

Based upon the literature, we developed a computational model of known interactions between essential immune cells (antigen-presenting macrophages, effector and regulatory T cells) and cytokine mediators (IL-2, TNFα, IFNγ) of granulomatous inflammation during sarcoidosis. The dynamics of these interactions are described by a set of ordinary differential equations. The model predicts bistable switching behavior which is consistent with normal (self-limited) and “sarcoidosis-like” (sustained) activation of the inflammatory components of the system following a single antigen challenge. By perturbing the influence of model components using inhibitors of the cytokine mediators, distinct clinically relevant disease phenotypes were represented. Finally, the model was shown to be useful for pre-clinical testing of therapies based upon molecular targets and dose-effect relationships.

**Conclusions/Significance:**

Our work illustrates a dynamic computer simulation of granulomatous inflammation scenarios that is useful for the investigation of disease mechanisms and for pre-clinical therapeutic testing. *In lieu* of relevant *in vitro* or animal surrogates, our model may provide for the screening of potential therapies for specific sarcoidosis disease phenotypes in advance of expensive clinical trials.

## Introduction

Sarcoidosis is a chronic granulomatous disease of unknown cause, for which relevant research models are lacking. Human research in sarcoidosis is hindered by the existence of diverse clinical phenotypes, presumably relating to genetic and environmental variables [1]. Genetic variability may also explain the unpredictable response to treatment among sarcoidosis patients [1]. Given the genetic diversity of the disease, environmental variables (e.g., antigen exposures) and the lack of relevant animal models, it would be necessary to recruit large numbers of patients, at a substantial cost, to represent all of the sarcoidosis phenotypes using conventional clinical research approaches. Alternatively, new generation, high-throughput genetic screening platforms provide an unprecedented opportunity to stratify the molecular basis of sarcoidosis disease phenotypes with the ultimate goal of individualizing therapy. To this end, it will be necessary to determine how genetic variability influences disease pathogenesis and treatment.

In this report, we focus on sarcoidosis phenotypes that are suspected to arise from defective antigen-dependent Th1 type immune responses associated with deregulated interactions among essential immune cells such as T effector cells, T regulatory cells, and antigen-presenting macrophages. The interactions among these cells are mediated by cytokines such as IL-2, IFNγ, and TNFα. We hypothesized that this complex interaction network contained sufficient information for the investigation of “normal” and “sarcoidosis-like” Th1 responses to antigens. Thus, we developed a computational model to represent the dynamics of this interaction network and its responses to perturbations. Our results are the first demonstration of an *in silico* model of granulomatous inflammation with potential applications for mechanistic and therapeutic research relating to sarcoidosis and other related diseases.

## Results

### A minimal model for Th1 activation

The hallmark of sarcoidosis is the preponderance of Th1 immune response to poorly characterized antigens. The differentiation of naive T cells upon antigen presentation and polarizing conditions has been the subject of previous mathematical modeling (see, for example, Yates *et al.* [2], Mariani *et al.* [3], and Mendoza *et al.* [4]). To cope with the complexity of the immune network, we first simplified these models to a minimal model that captures Th1 activation characterized by a bistable switch, as these previous models have shown. The following equation can describe this bistable switching behavior (without explicitly considering interactions with other immune cells for now):
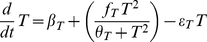
(1)


Note that we are interpreting T as Th1 activity or the density of CD4+ T effector cells (Teffs) with the Th1 phenotype. The first and last terms on the right-hand side of this equation set the net basal flux (influx rate, *β_T_*, minus the clearance rate, *ε_T_T*) of Teffs in the system. These terms give the basal steady state level of *T* equal to *β_T_/ε_T_*. The rate of Th1 activation is assumed to be given by the Hill-type function in the second term of Eqn 1. This sigmoidal function represents the autocatalytic increase in Th1 activity after antigen presentation and subsequent autocrine cytokine signaling which occurs for positive values of *f_T_*. As will be shown in the next subsection, the factor *f_T_* links Teffs to the influence of antigens (of density *A*) and cytokines (of density *c*); for now we use the following equation:
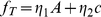
(2) where *η*_1_ and *η*_2_ are non-negative coefficients.

**Figure 1:**
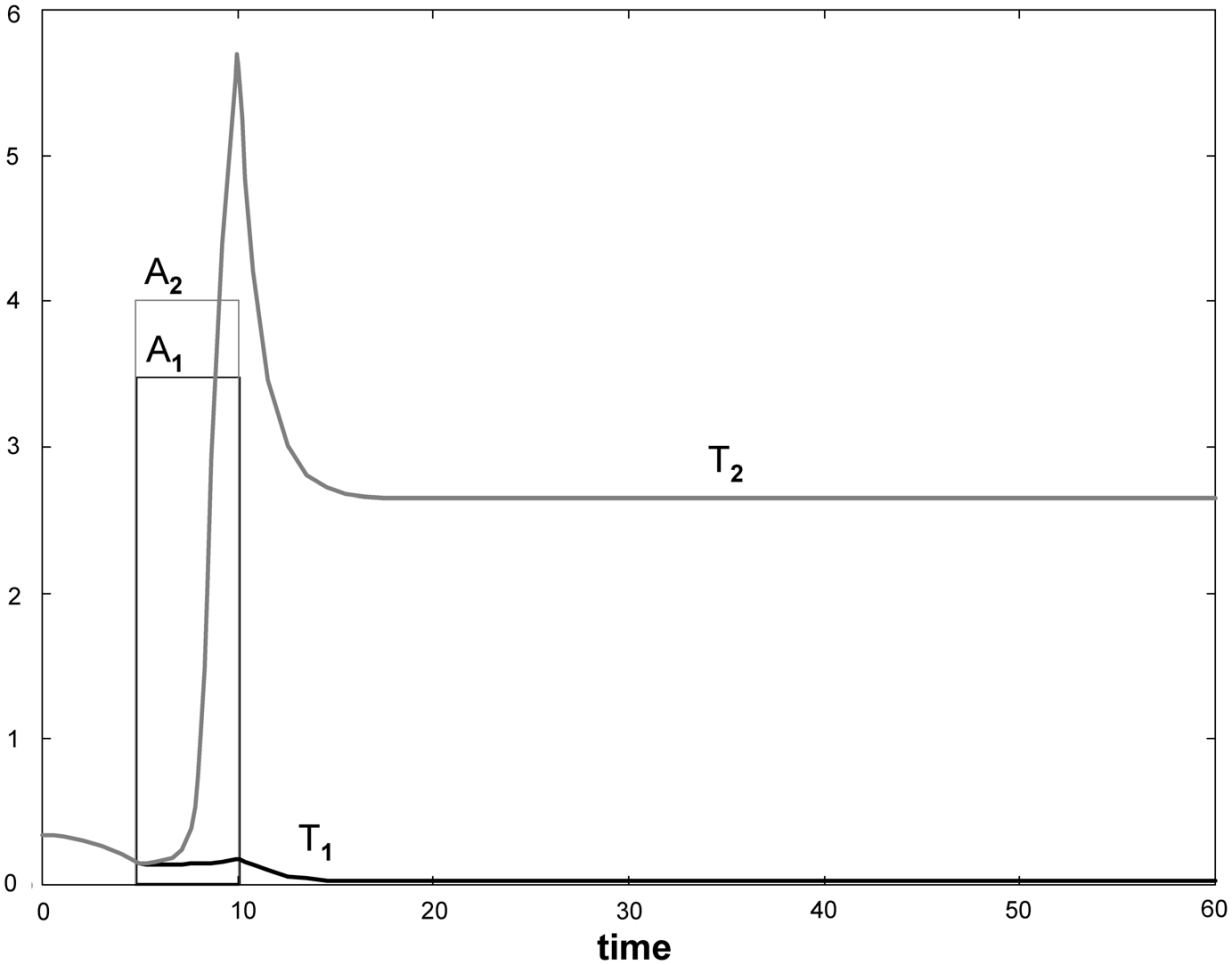
Bistable switching behavior of effector T cell (T) response. A simple model of the response of effector T cells to antigens and cytokines predicts a threshold of antigen level that triggers a Th1 immune response (see Eqns 1 and 2). *T* in Eqn 1 is interpreted as Th1 activity. A square pulse of antigen with amplitude A1 = 3.5 (applied from t = 5 to t = 10) fails to increase *T*, while increasing the amplitude to A2 = 4 leads to a rapid increase in Th1 activity at the level T2. Parameters: *θ*T = 1, *β*T = 0.02, *ε*T = 1, *c* = 3, *η*_1_ = *η*_2_ = 1, initial *T* = 0.34.

Supplementary information, represented by Eqn S1 and Figure S1 (see Text S1), proves that Eqn 1 gives rise to a bistable range of *f_T_*; in other words, within this range of *f_T_*, the system can be at low or at high Teff steady state activities, and which state is achieved depends on the initial conditions of the system. This bistability predicts that as *f_T_* is increased from a low value, there is a threshold value of *f_T_* where there occurs a discontinuous switch to high Th1 activity. In other words, sufficient increase in antigen and/or cytokine densities can bring about a sharp transition to high Teff steady state (*T_s_*) levels. Alternatively, the system can switch to the upper branch of steady states within the bistable range by perturbing *T* so that its value crosses the middle branch of the curve. This is illustrated in Figure 1 where a square pulse of *A* is applied to the system, and a pulse of sufficient amplitude succeeds in switching the system to a larger *T_s_*.

Regulatory T cells (Tregs) are expected to increase the threshold for achieving high steady state T activity. As per Eqn 1, an increase in the value of the parameter *θ_T_* in this simple model corresponds to an increase in Treg density in the detailed model discussed in the next subsection. This relationship is schematically represented in Figure S1 (see Text S1), wherein the effect of greater Treg activity (*θ_T_*) is to increase the value of *f_T_* where the switch to high *T_s_* activity occurs – that is, the antigen or cytokine threshold for promoting high-level Teff activity is increased.

### A model involving essential components of granulomas (Teffs, Tregs, and macrophages)

**Figure 2:**
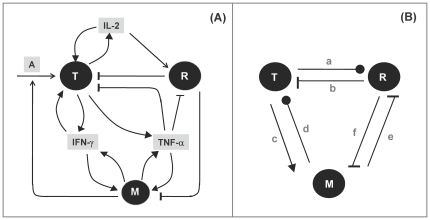
Modeling assumptions for Th1 responses. (A) The detailed immune network model involving Th1 activity (T), Tregs (R), and macrophages (M), with their interactions mediated by the cytokines IL-2, IFNγ, and TNFα. Arrows mean “upregulate” or activate while hammerheads mean “downregulate” or inhibit. A refers to the antigen presented by M to activate T. (Fig S2 in the Appendix shows a version of the network that corresponds to the differential equations of the model.) (B) A qualitative network (qNET) summarizing the pairwise interactions between the immune cells. Arrows ending with solid black circles represent either activation or inhibition. The edges a-f are explained in the text.

Here, we present a network model that involves the interactions of Teffs with macrophages and Tregs. The predisposition to sarcoidosis has implicated some dysfunction of naturally occurring, innate, Tregs [4]. Macrophages are also essential in the model because, first, they are known to be highly recruited by active Teffs to become part of granulomas (they represent the bulk of the granuloma by mass), macrophages synergize with Teffs in the secretion of IFNγ and TNFα, and macrophages interact with and affect Treg activity [5]–[9]. The model immune network shown in Figure 2A minimally depicts the essential interactions among Teffs, Tregs, macrophages, and key cytokines secreted by these cells that mediate cell-cell interactions, namely, IL-2, IFNγ and TNFα. As in the previous section, this model is phenomenological in that it does not include all known mechanistic details but rather captures the qualitative dynamics of the interactions among the players of the system – that is, how one player promotes or inhibits the densities or activities of other players in the network. Figure 2B summarizes the net interactions between cells in a so-called *qualitative network* (qNET), as we described previously [10]. As will be demonstrated in the next section, this qNET is useful to guide our intuition on the potential dynamics of the detailed model of Figure 2A.

For the model in Figure 2A, the rate of change of *T* is described by:
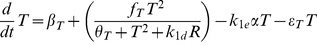
(3) where 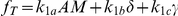


*A*, *M*, *δ* and γ are densities of antigens, macrophages, IL-2 and IFNγ, respectively. Note that the product *AM* represents antigen presentation to Teffs by macrophages. We assume that macrophages are the only antigen-presenting cells, and increasing *M* leads to increased antigen presentation to Teffs.

There are several mechanisms by which Tregs antagonize the activity of Teffs [9], [11], but we subsume all these mechanisms in the inhibitory effect of Tregs on the proliferation of Teffs as implemented by including *k_1d_R* in the denominator of the second term on the right-hand side of Eqn 3. Thus, the downregulation by Tregs of the production of IL-2, IFNγ and TNFα is indirectly caused by the inhibition of Teff proliferation.

The following equations for the rates of change in the densities of Tregs (*R*) and macrophages (*M*) can be understood from the interactions shown in Figure 2:
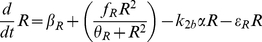
(4) where 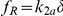


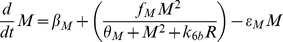
(5) where 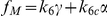


Also, looking at Figure 2, the rates of change in the densities of the cytokines can be described by the following differential equations:
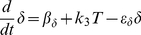
(6)

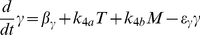
(7)

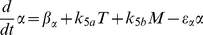
(8)


**Equations (3** **)–(8)** form the complete set of ordinary differential equations that describe the dynamics of our model (see Figure S2).

### Potential dynamics from the qNET structure

The qNET shown in Figure 2B summarizes the qualitative interactions between pairs of immune cells. This qNET can already give us some intuition as to what to expect for the long-term states of the system. Arrows mean “activate” or upregulate, while hammerheads mean “inhibit” or downregulate. Without Tregs, it is obvious that Teffs and macrophages synergize and activate each other, but only to a point where high levels of TNFα (a by-product of this synergy) are sufficient to suppress Teffs [this regulatory activity of macrophages towards Teffs has been suggested by Nagar *et al* [12] (see below)]. Without this macrophage-dependent suppression of Teffs, and without Tregs, a runaway immune response (unchecked activation and proliferation of Teffs and macrophages) is predicted.

Tregs exert suppressive activities on both Teffs and macrophages, as depicted through edges ***b*** and ***f***, respectively. The solid black circle at the end of edge ***a*** is initially an arrow (activation) representing the IL-2-dependent activation of Tregs by Teffs [13]. However this arrow may turn into a hammerhead (suppression) if the negative effects of TNFα (whose levels are also induced by Teffs) begin to dominate [12]. With edge **a** being an arrow, the pair of edges ***a*** and ***b*** is a negative feedback loop that could lead to an oscillation between the numbers of Teffs and Tregs (this oscillation could be damped or sustained, as previous mathematical modeling, and experimental observations, have shown [14], [15]. If edge ***a*** is a hammerhead, a toggle switch (mutual inhibition) could ensue; in other words, either Tregs annihilate Teffs or vice versa. Note that the interactions between Tregs and macrophages are also assumed to constitute a toggle switch (edges ***e*** and ***f***), based upon established mechanisms [16], [17].

What are the possible long-term state levels of Teffs, Tregs and macrophages as predicted by the qNET of Figure 2B? Using only a rough characterization of cellular levels (low and high, symbolized by 0 and +, respectively), not all of the 8 possible states can be realized due to the structure of the qNET, as shown in Table 1.

**Table 1:** Long-term states predicted by the qNET shown in Figure 2B.

	T	R	M
state 1 : healthy (Figs 3A, 6B, 6C)	0	0	0
state 2 : unlikely	0	+	0
state 3 : unlikely	0	0	+
state 4 :unlikely	0	+	+
state 5 :unlikely	+	0	0
state 6 : sarcoid, lymphopenia (Fig 5D)	+	+	0
state 7 : sarcoid (Figs 4A, 5B, 6A, 6E)	+	0	+
state 8 : sarcoid, lymphopenia (Figs 3C, 4B, 5A, 5C, 6D, 6F, 7A)	+	+	+

Zero (0) and plus (+) mean low and high values, respectively. Figure numbers under column 1 refer to examples found in simulations of the detailed model.

The ‘healthy state” (state 1) is characterized by very low or non-existent levels of Teffs, Tregs and macrophages. Possible “sarcoid states” are states 5–8, although state 5 is predicted to be unlikely from the qNET structure alone (because macrophages go up with Teffs in the absence of Tregs which is the only source of inhibition according to the qNET). Note that states 6 and 7 involve simultaneous high activities of Th1 and Tregs which, as will be explained in the next section, explains the immune paradox discussed by Miyara *et al* [4].

The important question that modeling hopes to contribute is to predict the possible scenarios on how a healthy state switches to a sarcoid state. For this, we need to perform computer dynamic simulations of the full model. The base parameters used for the detailed model (depicted in Figure 2A) are provided in Table 2.

**Table 2:** Base parameter values used for the detailed model in Fig. 2A (see also Fig. S2).

Initial Conditions	T = 0.01	R = 0.01	M = 0.001	δ = 0.001	γ = 0.001	α = 0.001		
Teff (T)	β_T_ = 0.05	ε_T_ = 1.0	θ_T_ = 1.0	k_1a_ = 1.0	k_1b_ = 20.5	k_1c_ = 10.0	k_1d_ = 1.0	k_1e_ = 0.1
Treg (R)	β_R_ = 0.05	ε_R_ = 1.0	θ_R_ = 1.0	k_2a_ = 1.0	k_2b_ = 0.1			
Macrophage (M)	β_M_ = 0.05	ε_M_ = 1.0	θ_M_ = 1.0	k_6_ = 1.0	k_6b_ = 1.0	k_6c_ = 0.1		
IL-2 (δ)	β_δ_ = 0.01	ε_δ_ = 10.0	k_3_ = 1.0					
IFNγ (γ)	βγ = 0.01	εγ = 10.0	k_4a_ = 1.0	k_4b_ = 1.0				
TNFα (α)	βα = 0.01	εα = 10.0	k_5a_ = 1.0	k_5b_ = 1.0				

### Thresholds of sarcoidosis (Th1 activation) related to amplitude of antigen exposure

**Figure 3:**
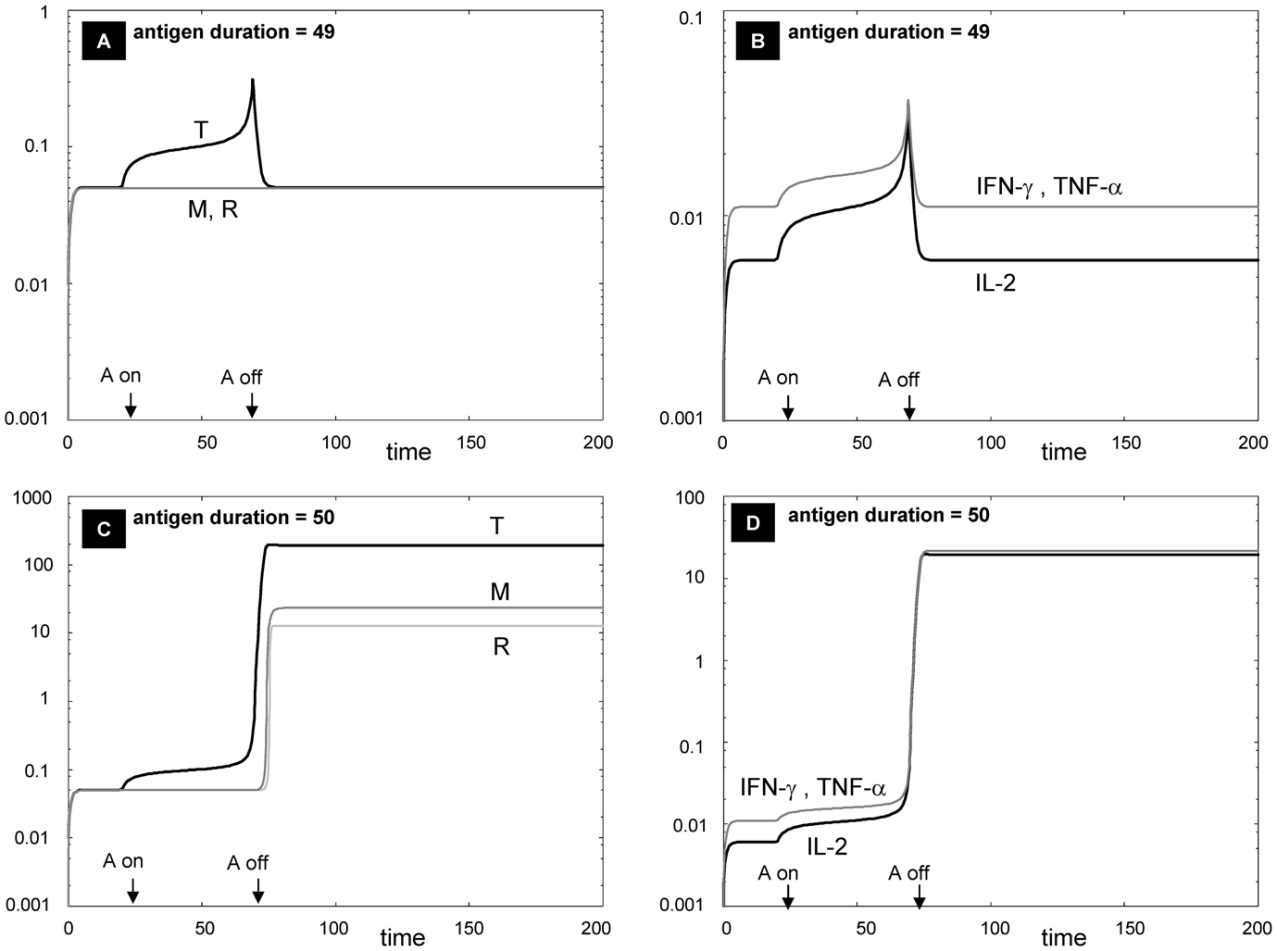
Antigen-dependency of Th1 activity. The detailed model of Fig 2A predicts thresholds of antigen level or duration that trigger Th1 activity. Shown in this figure are the dynamics of the model variables at pre- and post-threshold duration of antigen exposure. The system is first allowed to reach the low steady states of all variables, and then exposed to a square pulse of antigen (with amplitude A = 100) from t = 20 to t = 69 (A and B) or from t = 20 to t = 70 (C and D) as indicated by the arrows on the abscissa. All parameters as in Table 2, except k_2a_ = 2.1.

The typical immune response to a single antigen exposure is self-limited. However, antigen exposures of sufficient duration or amplitude are capable of inducing a new steady state characterized by dramatic expansion of immune cell populations along with sustained increases in pro-inflammatory cytokines (Figure 3). This scenario would correspond to persistent granulomatous inflammation (active sarcoidosis).

### Switching behavior of Treg levels due to sensitivity to IL-2

**Figure 4:**
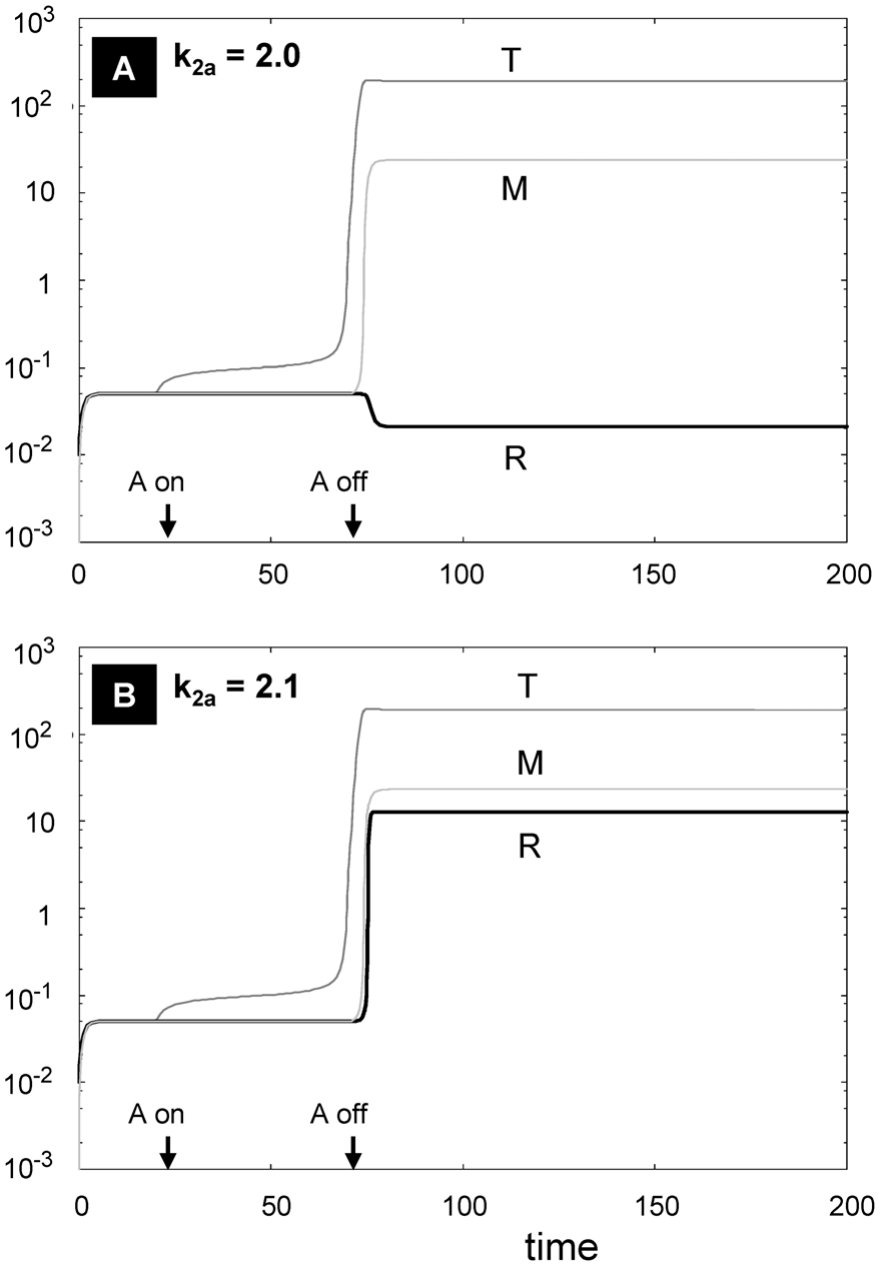
Sensitivity of Tregs (R) to the parameter k_2a_ (response to IL-2). The system is first allowed to reach the low steady states of all variables, and then exposed to a square pulse of antigen (with amplitude A = 100) from t = 20 to t = 70 as indicated by the arrows on the abscissa. All parameters as in Table 2, except k_2a_ which when increased above a specific threshold (compare Panel A *vs* Panel B) promotes a dramatic increase in Tregs.

Tregs are considered to be important determinants of granulomatous inflammation in patients with sarcoidosis [5], [18]. Based upon our modeling assumptions (Figure 2A) and previous reports [15], [19], it would appear that Treg activity is critically dependent upon IL-2. Figure 4 confirms that steady state level of Tregs sensitively depends on the parameter k_2a_ (which is the coefficient of the dependence of Treg proliferation on IL-2). Figure 4B corresponds to state 8 in Table 1, wherein Tregs are elevated along with Teff and macrophages. This represents the clinical scenario wherein active pulmonary sarcoidosis is paradoxically associated with anergy to new antigens (i.e., suppression by Tregs) [4].

### Modeling therapeutic interventions

**Figure 5:**
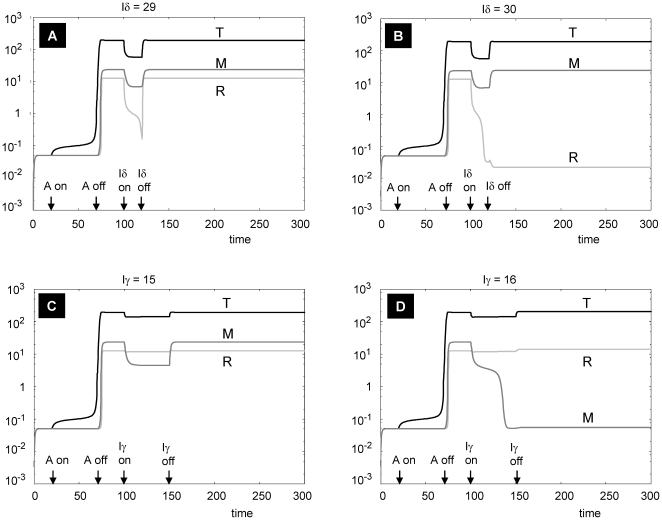
Single therapies using anti-IL2 (Iδ) or anti-IFNg (Iγ). The system is first allowed to reach low steady states of all variables, and then exposed to a square pulse of antigen (with amplitude A = 100) from t = 20 to t = 70 to obtain a sarcoid state. As shown in (A) and (B), the model predicts that there is a threshold of anti-IL2 (between amplitudes Iδ = 29 and Iδ =  30) that will push the steady state of R to a low level, but not T and M. The anti-IL2 therapy is represented by asquare pulse of Id given between t = 100 and t = 120. And as shown in (C) and (D), the model also predicts that there is a threshold of anti-IFNγ (between amplitudes Iγ = 15 and Iγ = 16) that will push the steady state of M to a low level, but not T and R. The anti-IFNγ therapy is represented by a square pulse of Iγ given between t = 100 and t = 150. All parameters as in Table 2, except k_2a_ = 2.1.

**Figure 6:**
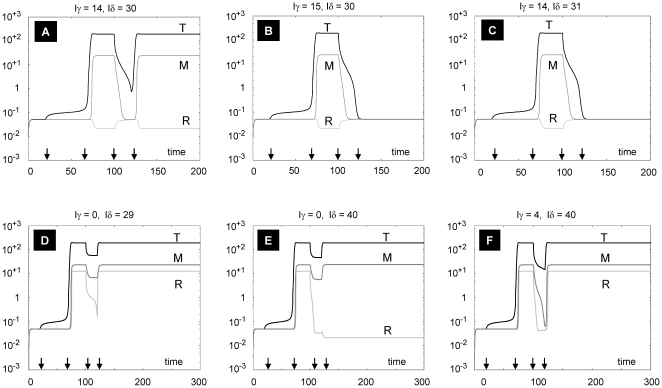
Combination therapies using anti-IL2 and anti-IFNγ. The system is first allowed to reach low steady states of all variables, and then exposed to a square pulse of antigen (with amplitude A = 100) from t = 20 to t = 70 to obtain a sarcoid state. For (A)-(C), k_2a_ = 2.0. Combinations of anti-IFNγ (Iγ) and anti-IL2 (Iδ) are applied as square pulses at the same time, between t = 100 and t = 120. The combination in (A) is not successful, while those of (B) and (C) are successful in pushing down the levels of T and M (although there is a slight increase in R compared to its level in (A), R is still very low). For (D)–(F), k_2a_ = 2.1. An increase in anti-IL2 (Iδ) from 29 to 40 brings down R, but not T and M. Note that a small addition of Iγ as shown in (F) returns R to the high steady state level. All parameters as in Table 2, except k_2a_.

Assuming no change in Treg sensitivity, as modeled in Figure 4, we sought to determine the effects of suppression of either IL-2 or IFNγ levels (corresponding to anti-IL-2 or anti- IFNγ therapy). As shown in Figure 5, “monotherapy” with either anti-IL-2 or anti- IFNγ is capable of suppressing Treg and macrophage activation, respectively, without affecting high steady-state activation of Teff cells. In contrast, Figure 6 shows that combinations of anti-IL-2 and anti- IFNγ at appropriate “doses” (as represented by the amplitude of inhibition) maintains all cell lines in the low steady-state activation state following antigen challenge (i.e., the normal response). Thus, assuming that the modeling conditions accurately depict the mechanisms regulating granuloma formation in the setting of sarcoidosis, careful titration of combination therapy with anti-IL-2 and anti-IFNγ is predicted to effectively attenuate disease activity.

### Anti-TNFα therapy can promote the sarcoidosis phenotype

**Figure 7:**
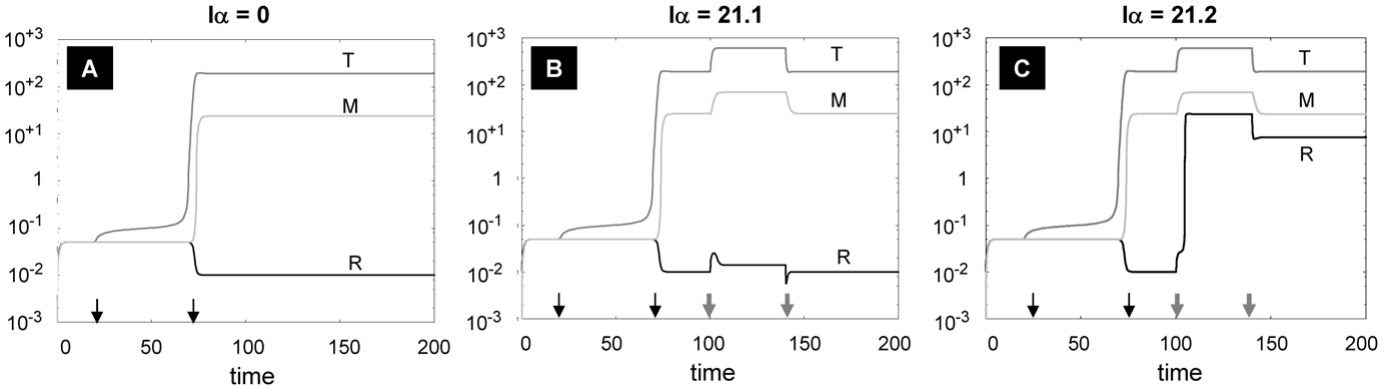
Single therapy using anti-TNFα (Iα). The system is first allowed to reach low steady states of all variables, and then exposed to a square pulse of antigen with amplitude A = 100 from t = 20 to t = 70 (indicated by the first two black arrows from the left) to obtain a sarcoid state. (A) Without anti-TNFα (Iα = 0). (B) Anti-TNFα is introduced as a square pulse with amplitude Iα = 21.1 from t = 100 to t = 140 (indicated by the last pair of thick gray arrows); (C) Anti-TNFα is introduced as a square pulse with amplitude Ia = 21.2 from t = 100 to t = 140 (indicated by the last pair of thick gray arrows). All parameters as in Table 2, except k_2a_ = 2.1 and k_2b_ = 0.2.

Considering that sarcoidosis is a polygenic disease with diverse phenotypes, models must be adjusted to represent each disease phenotype. Whereas anti-TNFα therapy often suppresses sarcoidosis disease activity [20], [21], one clinical variant of sarcoidosis is actually promoted by anti-TNFα therapy [22], [23]. In this regard, it is interesting that the current model assumptions predict that monotherapy with anti-TNFα would either be ineffective or could paradoxically promote the sarcoidosis state (Figure 7).

## Discussion

*In silico* modeling of lung disease is in its infancy, as reflected by the few published attempts to date, particularly relating to lung physiology [24], [25]. However, leading scientific organizations like the National Institutes of Health (NIH) anticipate the need for *in silico* modeling to accommodate the exponential growth of information emerging from human genetic studies. With respect to strategic planning towards the goal of providing “personalized medicine” based upon genetic profiling, the NIH seeks to “use *in silico* and statistical modeling/simulation strategies to design and inform genetic/pharmacogenetics clinical trials, so results can eventually be used for tailoring treatment or prevention” (http://www.nhlbi.nih.gov/about/dcvd/sp/dcvd-sp-goal-1.1.htm). The *in silico* sarcoidosis model presented here possesses relevant features, including representation of “normal” and “disease” phenotypes, and the capacity to perform preclinical therapeutic testing. As such, this model serves as a promising template for future sarcoidosis research.

Based upon putative interactions among critical components essential for granuloma formation, our model demonstrates that a biphasic immune response is expected depending on the nature of the antigenic challenge. As shown in Figure S1, suppression of Treg function, as was demonstrated in the lungs of sarcoidosis patients [4] presumably in response to TNFα [9], is sufficient to reduce the threshold for a sustained antigen-dependent Th1 response. *In lieu* of an altered immune response (i.e., normal host), the model further predicts that a sarcoidosis-like Th1 response could be elicited by an antigen challenge of sufficient intensity. This model behavior is consistent with a number of clinical reports of previously healthy patients developing sarcoidosis following a single, massive antigen exposure, such as the case in first-responders to the 9/11 disaster in New York City [26], [27].

To the extent that the computational model represents mechanisms predisposing to unregulated granuloma formation in the context of sarcoidosis, various “treatments” can be simulated by mathematically manipulating one or more of the extracellular immunomodulating molecules. This approach is illustrated in Figures 5, 6 and 7, wherein suppression of any single molecule (IL-2, IFNγ or TNFα) fails to decrease Teff density in the sarcoidosis phenotype, whereas simultaneous suppression of IL-2 and IFNγ restores the system to the baseline state (i.e., disease remission). These results are strongly dependent on the modeling assumptions, including the functional status of specific components of the network, as represented by changes in the kinetics or the character (inhibition, activation) of the interactions of one or more of the components in Eqns 3–8. A key feature of our model is that it conveniently allows for adjustments to accommodate diverse disease mechanisms. As a specific example, the sarcoidosis-associated *BTNL2* mutation which results in a truncation mutation of the BTNL2 protein, a B7-family protein predicted to inhibit interactions between antigen-presenting cells and Teffs [28], would be represented by an increase in *f_T_* in Eqn 3. This would have the same effect as diminishing Treg function (*θ_T_*, Eqn 3) in that the threshold for achieving the high *Ts* would be reduced, favoring the sarcoidosis phenotype.

Interestingly, our model suggests a resolution of the clinical paradox discussed by Miyara *et al* [4], wherein excessive antigen-mediated inflammation in the lungs coexists with impaired antigen responsiveness (anergy) elsewhere. Recent studies suggest that expansion of Tregs at the sites of active granulomatous tissue inflammation causes suppression of T cell proliferation elsewhere, leading to peripheral lymphopenia and anergy, which are well-documented manifestations of active pulmonary sarcoidosis [29], [30]. This scenario is represented by conditions 6 and 8 in Table 1, and Figures 4B, 5C, 6D, 6F, 7C.

Admittedly, our model is incomplete in that detailed information relating to the actual kinetics and functions of the system components are not well characterized, and many ancillary cells and molecules are not considered. For instance, recruitment of macrophages induced by IL-2 is not included in model [31] and many cells and cytokines involved in granuloma formation (e.g., natural killer T cells, α/δ T cells, IL-12, IL-23 [8], [32]) are not considered. Nonetheless, the qualitative framework of the model is adaptable as more details relating to mechanisms of granuloma formation and disease pathogenesis emerge, including the effects of genetic variability. Moreover, model simulations are convenient in that they can be run using inexpensive and readily-available computational facilities, as well as practical in that they accurately portray relevant clinical phenotypes, even in the simplified form presented herein.

In summary, we provide the first dynamic computer simulation of granuloma formation in sarcoidosis, a disease for which relevant *in vitro* or animal models do not currently exist. The qualitative network is shown to accurately model distinct disease mechanisms, including a sarcoidosis state resulting from either massive antigen exposure or altered immune (e.g., Treg) function, and specific sarcoidosis phenotypes (e.g., lymphopenic sarcoidosis). The model demonstrates the capacity for pre-clinical therapeutic testing, and adaptability based upon distinct disease immunophenotypes, such as those dictated by genetic variability.

## Methods

To determine the steady states of the detailed model, the baseline values for each parameter was set to the values shown in Table 2 and further modified, as described for each experimental condition, and the corresponding systems of differential equations (Eqns 3–8, above) were solved numerically using the BerkeleyMadonna software (http://berkeleymadonna.com).

## Supporting Information

Figure S1 **Steady state of T (** ***T*** **s) as a function of** ***fT*** **(from** **Eqn 2** **) for two values of θT (θT = 1 for the black curve, and θT = 0.5 for the gray curve).** Other parameter values: *bT* = 0.05, *eT* = 1.
(TIF)

Figure S2 **The detailed immune network model, showing numbered interactions representing the model differential equations for each cell [T effector (T), Treg (R), Macrophage (M)] or molecule (TNFα, IFNγ, IL-2), represented by** **Eqns 3** **–** **8** **.** Arrows mean “upregulate” or activate while hammerheads mean “downregulate” or inhibit.
(TIF)

Text S1 **Supporting information demonstrating a bistable switch between low and high Teff steady states predicted by the model.**
(DOC)
