# Supplementary figures and images for: An In Silico Modeling Approach to Understanding the Dynamics of Sarcoidosis
Source: PLoS One. 2011 May 27;6(5):e19544. doi: 10.1371/journal.pone.0019544 (PMC3103504; doi:10.1371/journal.pone.0019544)

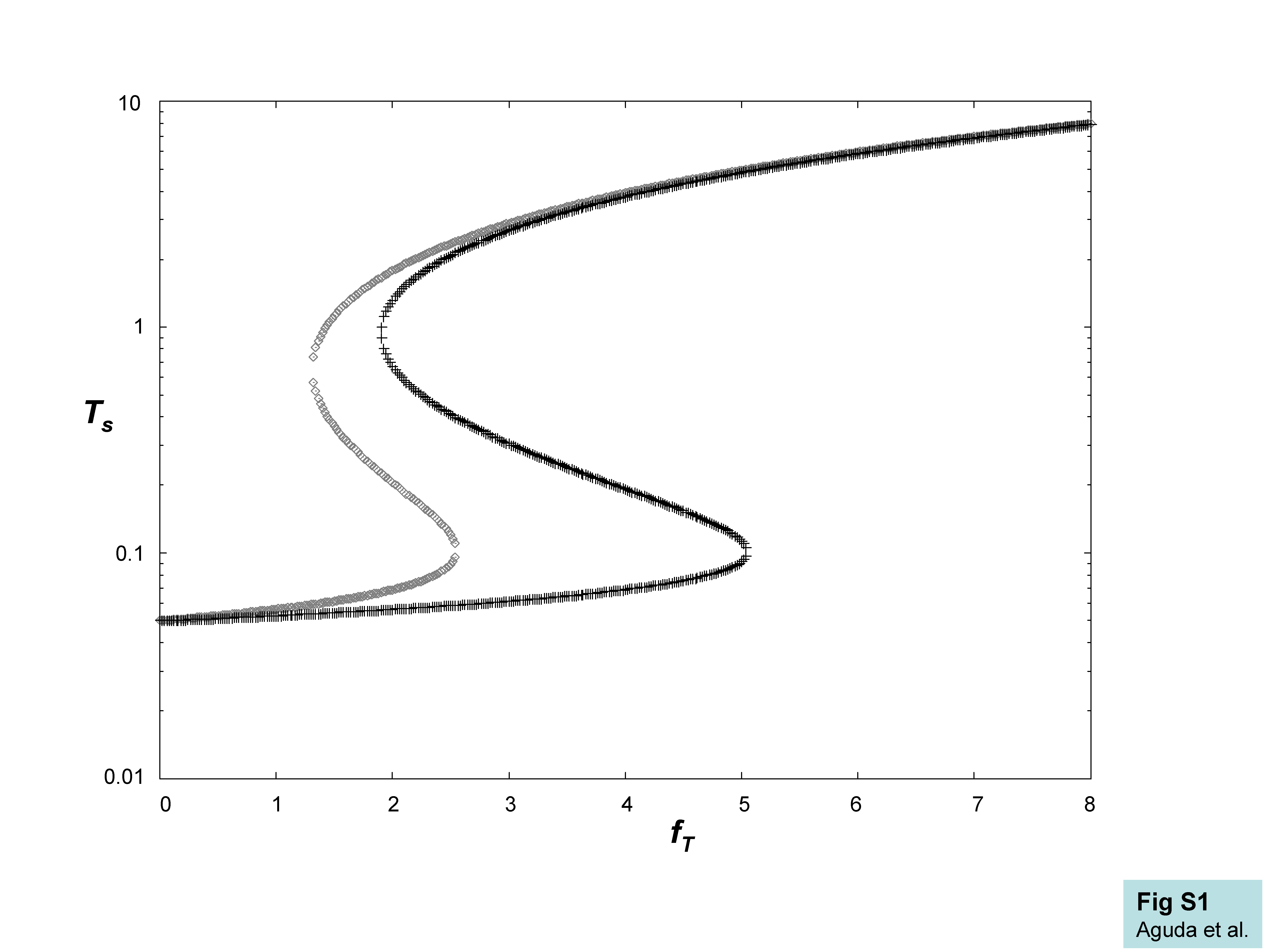

Supplement: Figure S1 — Steady state of T ( T s) as a function of fT (from Eqn 2 ) for two values of θT (θT = 1 for the black curve, and θT = 0.5 for the gray curve). Other parameter values: bT = 0.05, eT = 1. (TIF) [file pone.0019544.s001.tif]

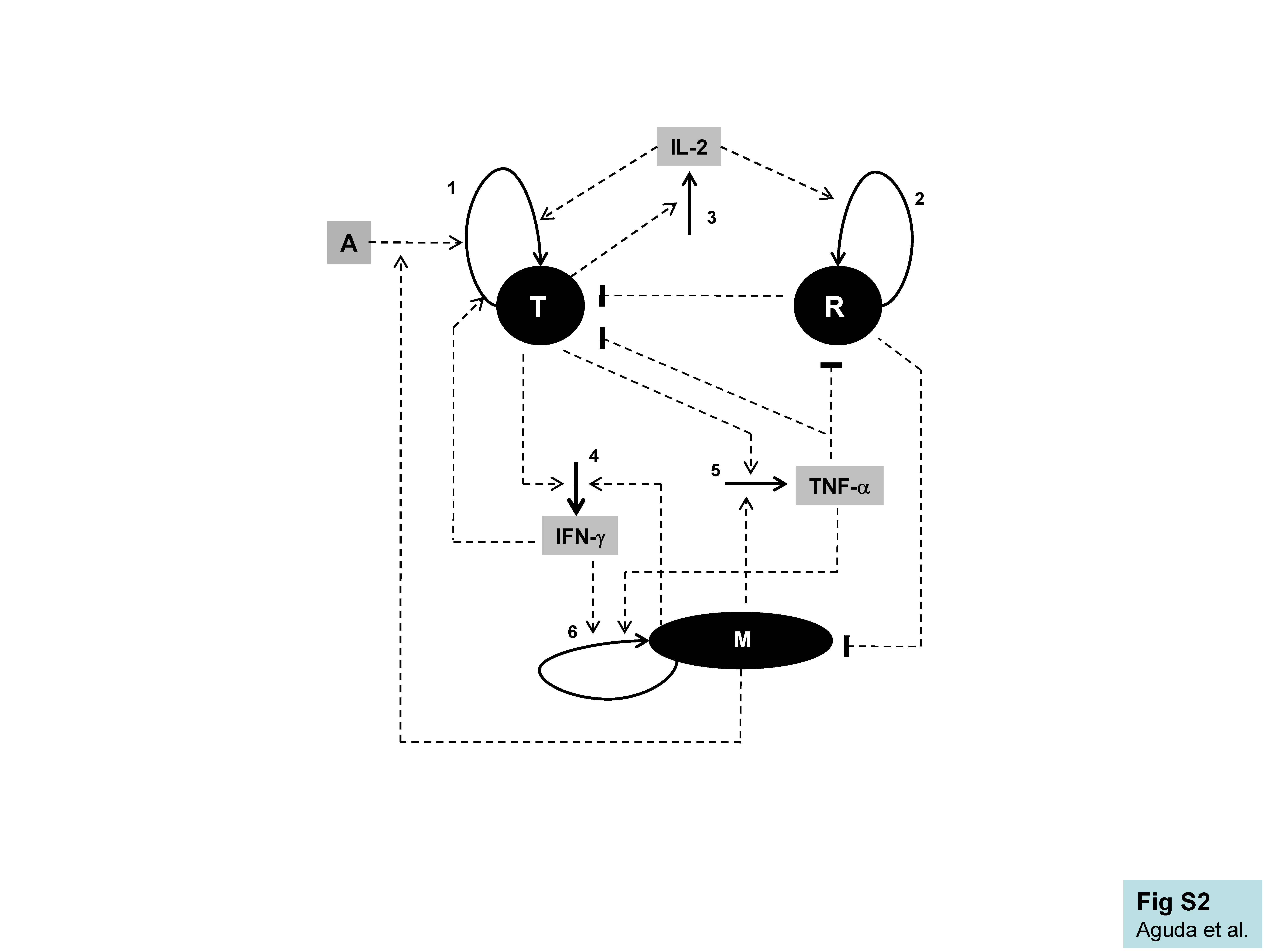

Supplement: Figure S2 — The detailed immune network model, showing numbered interactions representing the model differential equations for each cell [T effector (T), Treg (R), Macrophage (M)] or molecule (TNFα, IFNγ, IL-2), represented by Eqns 3 – 8 . Arrows mean “upregulate” or activate while hammerheads mean “downregulate” or inhibit. (TIF) [file pone.0019544.s002.tif]
