# Supplementary material for: An In Silico Modeling Approach to Understanding the Dynamics of Sarcoidosis
Source: PLoS One. 2011 May 27;6(5):e19544. doi: 10.1371/journal.pone.0019544 (PMC3103504; doi:10.1371/journal.pone.0019544)
Supplement: Text S1 — Supporting information demonstrating a bistable switch between low and high Teff steady states predicted by the model. (DOC) [file pone.0019544.s003.doc]

**Text 1**

Supporting information demonstrating a bistable switch between low and high Teff steady states predicted by the model.

The steady states of Teffs (referred to as *T_s_* and obtained from *dT/dt* = 0) are given by the positive roots of the following cubic polynomial

**(S1)** $\varepsilon_{T}T_{s}^{3}-(f_{T}+\beta_{T})T_{s}^{2}+\varepsilon_{T}\theta_{T}T_{s}-\beta_{T}\theta_{T}=0$

The steady states, *T_s_*, as *f_T_* (i.e., the effects of antigens and/or cytokines; see Eqn 2) is increased are shown in Fig S1 for two values of *θ_T_*. For each graph, there is a range of *f_T_* where 3 steady states coexist for a fixed set of parameters. This is called the bistable range because there are two stable steady states (the top and bottom steady states); the steady states in the middle branch are unstable. Thus, Eqn S1 captures the bistable switch predicted by the modeling performed by Yates, Callard & Stark [2] on the differentiation of naïve T cells to Th1 or Th2 helper cells. If *f_T_* increases from very small values (e.g., to the left of the left knee of the curves in Figure S1), the system will trace the lowest branch of steady states until it reaches the right knee of the curve where a switch to high *T_s_* occurs (dashed vertical arrow). In other words, sufficient increase in antigen and/or cytokine densities can bring about a sharp transition to high *T_s_* levels.
